# Topical mitomycin‐C as an adjuvant to multimodal endoscopic treatment for tracheobronchial stenosis secondary to endobronchial tuberculosis

**DOI:** 10.1002/rcr2.711

**Published:** 2021-01-25

**Authors:** Nai‐Chien Huan, Khai Lip Ng, Mona Zaria Nasaruddin, Noorul Afidza Muhammad, Ummi Nadira Daut, Jamalul Azizi Abdul Rahaman

**Affiliations:** ^1^ Department of Pulmonology Serdang Hospital Kajang Malaysia; ^2^ Department of Medicine Universiti Putra Malaysia Kajang Malaysia

**Keywords:** Bronchomalacia, bronchoscopy, endobronchial tuberculosis, mitomycin‐C, tracheobronchial stenosis

## Abstract

Tracheobronchial stenosis secondary to endobronchial tuberculosis (TSTB) is a rare but debilitating complication of endobronchial tuberculosis (EBTB). Topical mitomycin‐C (TMC) has been successfully utilized to restore airway patency and to prevent recurrence of TSTB, although little is known about its exact efficacy. Here, we report the biggest case series to date involving seven patients who received TMC as part of multimodality endoscopic treatment for TSTB with varying levels of success. All patients presented with dyspnoea during or after treatment completion for pulmonary tuberculosis (PTB). Four patients had short‐segment concentric membranous TSTB while two patients had concurrent bronchomalacia. Another one patient had a thick fibrotic band adjacent to luminal opening. We hypothesize that TMC is more efficacious in short membranous stenosis without concurrent bronchomalacia and/or thick fibrotic bands. More studies are needed to bridge the current gaps in knowledge regarding the optimal role and benefits of TMC for TSTB patients.

## Graphical abstract

Tracheobronchial stenosis secondary to endobronchial tuberculosis is a debilitating disease but its optimal treatment strategy remains undefined. With a good safety profile, topical mitomycin‐C (TMC) appears to be a useful adjuvant to endoscopic treatment for tuberculosis‐related airway stenosis. This case series aims to highlight the current gaps in knowledge regarding the optimal role of TMC and whether certain airway features such as bronchomalacia or thick fibrotic band can influence its efficacy.

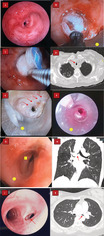

## Introduction

Endobronchial tuberculosis (EBTB) is defined as tuberculous infection involving the tracheobronchial tree with histopathological and/or microbial evidence [1]. Tracheobronchial stenosis secondary to endobronchial tuberculosis (TSTB) is a rare but debilitating and potentially life‐threatening form of EBTB, necessitating airway intervention such as surgery or endoscopic procedures. Endoscopic interventions including balloon dilatation, mechanical coring, laser recanalization, cryo‐ablation, and airway stent insertion are acceptable measures to attain meaningful symptomatic relief and to improve quality of life among patients with TSTB [2, 3, 4, 5]. Topical mitomycin‐C (TMC), a chemotherapeutic agent, has been used to restore airway patency in TSTB patients [6, 7, 8]. However, little is known about the exact role, duration of benefits, and whether certain airway characteristics such as bronchomalacia, presence of thick fibrotic band, and/or length of stenosis will influence the efficacy of TMC. Here, we report the biggest case series to date involving seven patients who received TMC as part of multimodality endoscopic treatment for TSTB with varying levels of success (Table 1).

**Table 1:** Clinical characteristics and outcomes of patients with TSTB.

Case number	Case 1	Case 2	Case 3	Case 4	Case 5	Case 6	Case 7
Age (years), gender	32, Female	57, Female	38, Female	26, Male	43, Female	29, Female	26, Female
Comorbidities	Nil	Nil	Nil	Nil	Dyslipidaemia, gastro‐oesophageal reflux disease	Nil	Gut tuberculosis 2014
Year of PTB	2020	2018	2010	2016	2009	2017	2019
Year of onset of symptoms	2020 (while on PTB treatment)	2019 (after the completion of PTB treatment)	2017	2017 (while on PTB treatment)	2019	2019 (a year after the completion of PTB treatment)	2019 (while on PTB treatment)
Symptoms	Dyspnoea, wheezing, orthopnoea	Recurrent dyspnoea, stridor	Dyspnoea on exertion	Dyspnoea, cough, weight loss	Dyspnoea, cough	Dyspnoea	Dyspnoea, reduced effort tolerance
CT findings	Luminal narrowing of upper one‐third of trachea, distal airways patent	Luminal narrowing of mid‐trachea, distal airways patent	Narrowed segment at right main bronchus, distal airways patent	Multilevel stenosis of trachea and right main bronchus with right upper lobe collapse	Tracheal stenosis at T3 and T4 levels and right main bronchus stenosis, distal airways patent	Left main bronchus stenosis with left upper lobe collapse	Multilevel stenosis of trachea, left and right main bronchus
Bronchoscopy findings	Tracheal stenosis, luminal opening 2 mm, length of stenosis 0.5 cm	Tracheal stenosis, luminal opening 2 mm, length of stenosis 1.5 cm	Stenosed and malacic right main bronchus, luminal opening 3 mm, length of stenosis/bronchomalacia 2 cm	Tracheal stenosis (able to pass through scope), right main bronchus stenosis, luminal opening 2 mm, length of stenosis 0.6 cm	Right main bronchus stenosis, luminal opening 2 mm, length of stenosis approximately 1 cm	Bronchomalacia of left main bronchus, length 2 cm, stenosis at distal end of left main bronchus, luminal opening 3 mm	Tracheal stenosis (able to pass through scope), right main bronchus totally obstructed, left main luminal opening 3 mm
Bronchomalacia	No	No	Yes	No	No	Yes	No
Thick fibrotic band	No	Yes	No	No	No	No	No
Membranous concentric stenosis	Yes	No	No	Yes	Yes	No	Yes
Intervention performed	Balloon dilatation and TMC application over trachea	Balloon dilatation, mechanical coring of stenotic segment and then TMC application over trachea	Balloon dilatation and TMC application over right main bronchus	Balloon dilatation and TMC application over right main bronchus	Balloon dilatation and TMC application over right main bronchus	Balloon dilatation and TMC application over left main bronchus	Balloon dilatation and TMC application over trachea and left main bronchus
Procedure‐related complication	Nil	Nil	Nil	Nil	Small bronchial tear during balloon dilatation (<0.5 cm), managed conservatively	Nil	Small tracheal tear during balloon dilatation (<0.5 cm), managed conservatively
TMC‐related complication	Nil	Nil	Nil	Nil	Nil	Nil	Nil
Outcome	Successful procedure with symptomatic relief at four months during the last encounter	Initially successful but rapid symptom recurrence within two weeks	Initially successful but rapid symptom recurrence in two weeks	Successful procedure with symptomatic relief for a year before recurrence	Successful procedure with symptomatic relief at 15 months during the last encounter	Initially successful but developed recurrence of symptoms after two months. Repeated scope showed restenosis	Successful procedure with symptomatic relief at five months during the last encounter
Further treatment	For surveillance bronchoscopy	Successful tracheal resection surgery	Scheduled for silicone stent insertion	Underwent two further sessions for balloon dilatation and TMC in 2018 and 2020	For surveillance bronchoscopy	Conservative management as patient declined further treatment	For surveillance bronchoscopy

CT, computed tomography; PTB, pulmonary tuberculosis; TMC, topical mitomycin‐C; TSTB, tracheobronchial stenosis secondary to endobronchial tuberculosis.

## Case Series

### Case 1

**Figure 1:**
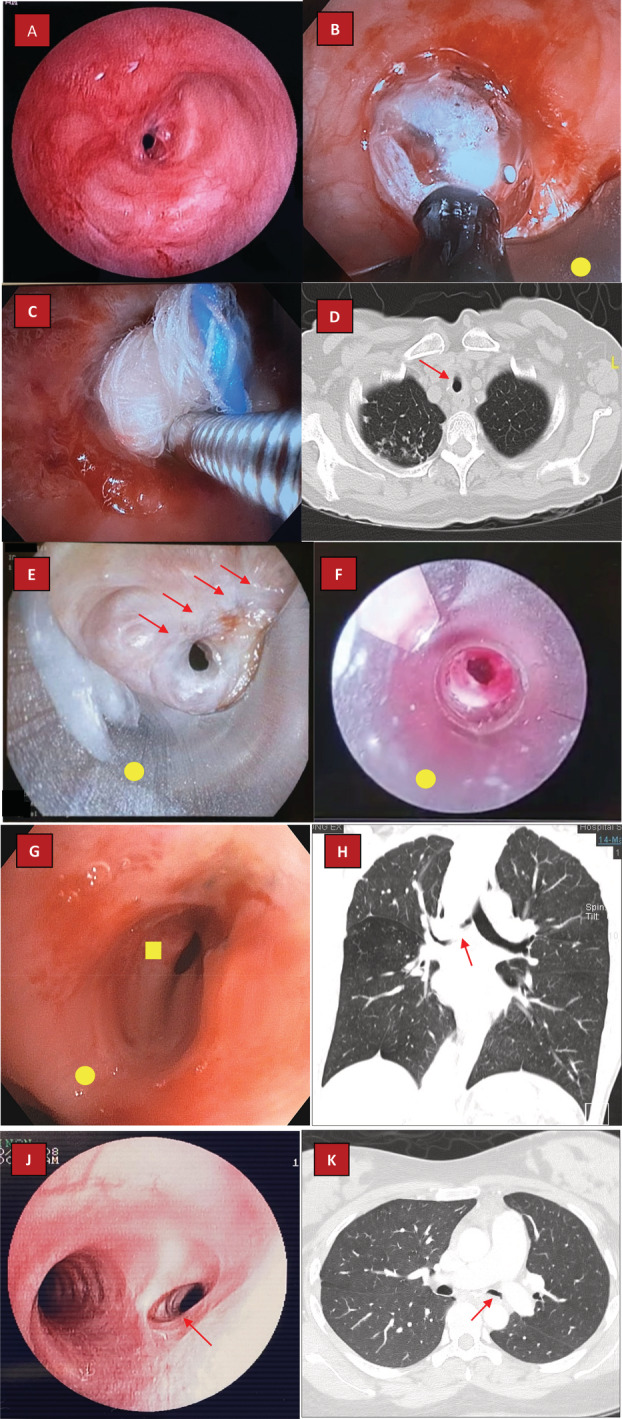
Computed tomography (CT) and bronchoscopy images. (A) Flexible bronchoscopy demonstrating short‐segment concentric membranous tracheal stenosis with opening of approximately 2 mm. (B) Attempting balloon dilatation of stenotic segment under rigid bronchoscopy (bevel of rigid bronchoscope marked with yellow circle). (C) Application of topical mitomycin‐C (TMC) at stenotic segment using soaked gauze balls post balloon dilatation. (D) CT demonstrating stenosis at mid‐trachea (red arrow) due to endobronchial tuberculosis (EBTB). (E) Bronchoscopy demonstrating severe tracheal stenosis with opening of 2 mm. A dense, pale, and thick fibrotic band (red arrows) was seen immediately anterior to the luminal opening. Bevel of rigid bronchoscopy marked with yellow circle. (F) Attempting mechanical coring and dilatation of tracheal stenosis by using a rigid bronchial tube (yellow circle). (G) Bronchoscopy showing bronchomalacia and stenosis of the right main bronchus (yellow square); main carina marked with yellow circle. (H) Right main bronchus stenosis (red arrow) as demonstrated on CT. (J) Surveillance bronchoscopy 15 months post balloon dilatation and TMC showing a narrowed but patent right main bronchus (red arrow). Right main bronchus was severely stenosed with a luminal opening of 2 mm pre procedure. The patient remained well. (K) CT showing a narrowed left main bronchus due to EBTB (red arrow).

A 32‐year‐old lady with smear‐positive pulmonary tuberculosis (PTB) on treatment presented with recurrent episodes of shortness of breath (Modified Borg Dyspnoea Scale 6 at rest: severe) and orthopnoea. She was treated as presumed adult‐onset asthma by a primary care doctor and was empirically commenced on inhalers but to no avail. Computed tomography (CT) demonstrated luminal narrowing of the upper one‐third of trachea secondary to EBTB. Bronchoscopy confirmed the presence of a concentric membranous tracheal stenosis with an opening diameter of 2 mm and a length of 0.5 cm (Fig. 1A). As her tracheal stenosis was beyond the subglottic level, after discussion with colleagues from ear‐nose‐throat (ENT) team, she underwent rigid bronchoscopy followed by gradual balloon dilatation of stenotic segment (Fig. 1B). TMC was subsequently applied using soaked gauze balls at a dose of 0.4 mg/mL for 2 min each over upper, middle, and lower parts of the stenotic segment (Fig. 1C). Post procedure, she reported improvement in symptoms (Modified Borg Dyspnoea Scale 4 at rest: moderate) which were sustainable at four months during her last clinical encounter without any TMC‐related adverse events.

### Case 2

A 57‐year‐old lady with PTB two years previously presented with dyspnoea and stridor for the past year (Modified Borg Dyspnoea Scale 7 at rest: very severe) due to TSTB. Prior to this, she underwent multiple endoscopic procedures (balloon dilatation and laser cauterization) at a private hospital. CT demonstrated luminal narrowing at mid‐trachea (Fig. 1D). The stenotic segment length was at 1.5 cm, significantly longer than the first case. Flexible bronchoscopy confirmed tracheal stenosis with an opening diameter of 2 mm. A dense and thick fibrotic band was seen anterior to the luminal opening, presumably aggravated by scarring secondary to previous procedures (Fig. 1E). She underwent rigid bronchoscopy followed by balloon dilatation of stenotic segment. Following that, mechanical coring of stenotic segment was carefully performed by utilizing a rigid bronchial tube (outer diameter 10 mm, colour coded red, Series‐2 Efer‐Dumon Bronchoscope, France) (Fig. 1F). TMC was then applied using soaked gauze balls at a dose of 0.4 mg/mL for 2 min each over upper, middle, and lower parts of the stenotic segment. Post procedure, she initially reported symptomatic improvements (Modified Borg Dyspnoea Scale 5 at rest: moderate) which lasted for less than two weeks. She subsequently successfully underwent airway surgery for resection of stenotic segment.

### Case 3

A 38‐year‐old lady with PTB 10 years previously presented with a three‐year history of progressively worsening dyspnoea (Modified Borg Dyspnoea Scale 3 at rest: moderate) due to right main bronchus stenosis. Flexible bronchoscopy confirmed a narrowed and malacic right main bronchus with a length of approximately 2 cm and an opening diameter of 3 mm (Fig. 1G). Balloon dilatation of the diseased segment was done via rigid bronchoscopy followed by application of TMC. Post procedure, she reported dramatic improvements in symptoms which lasted for just two weeks. Repeated bronchoscopy showed recurrence of bronchomalacia. Following that, a multidisciplinary discussion involving cardiothoracic surgeons, pulmonologists, thoracic radiologists together with patient herself and her family members was conducted to determine the next best management plans. Airway surgery was deemed risky due to long diseased segment making end‐to‐end anastomosis technically challenging. As the patient was keen to proceed with intervention, we did not pursue further with regards to conservative measures such as continuous positive airway pressure (CPAP). She was subsequently scheduled for silicone stent insertion at right main bronchus.

### Case 4

Back in December 2016, a 22‐year‐old gentleman presented with cough, weight loss, and persistent dyspnoea on exertion (Modified Borg Dyspnoea 4 at rest: somewhat severe) despite already on three months of medications for PTB. CT revealed multilevel stenosis involving the trachea and right main bronchus with concurrent right upper lobe collapse (Fig. 1H). Flexible bronchoscopy confirmed a stenosed right main bronchus without bronchomalacia or thick fibrotic band. Rigid bronchoscopy was performed along with gradual balloon dilatation of the stenotic segment before the application of TMC. Again, TMC was applied via soaked gauze balls for a total of eight times along the diseased airway. He reported significant symptomatic relief which lasted for more than a year before recurrence. As he rejected surgery, he subsequently underwent another two rounds of rigid bronchoscopy, balloon dilatation, and TMC (April 2018 and January 2020) without any adverse events. Latest surveillance scope by June 2020 confirmed a patent right main bronchus. He remained well and active in between procedures.

### Case 5

A 43‐year‐old lady with PTB 10 years ago presented with a two‐year history of cough and worsening dyspnoea (Modified Borg Dyspnoea 5 at rest: severe). Work‐up for recurrence of PTB was negative but CT showed tracheal narrowing with right main bronchus stenosis. Flexible bronchoscopy revealed a short‐segment concentric membranous stenosis at right main bronchus with an opening diameter of 2 mm. Similar to other cases, she underwent rigid bronchoscopy for balloon dilatation of the right main bronchus before TMC application for a total of six times along the airway. Her procedure was complicated by a small airway tear (<0.5 cm) at the right main bronchus but she nevertheless remained stable without needing emergency airway surgery. She reported sustained improvements in dyspnoea post procedure (Modified Borg Dyspnoea 2: slight). Her latest surveillance scope done more than 15 months post TMC showed a narrowed but patent right main bronchus (Fig. 1J).

### Case 6

A 29‐year‐old lady with a history of PTB back in 2017 presented with worsening dyspnoea (Modified Borg Dyspnoea Scale 3 at rest: moderate) a year after the completion of anti‐tuberculosis chemotherapy. Similar to the first case, she was initially empirically treated (unsuccessfully) as presumed adult‐onset asthma. CT showed left main bronchus stenosis with segmental atelectasis of the left upper lobe (Fig. 1K) while bronchoscopy revealed bronchomalacia with fibrostenosis at the distal end of left main bronchus. Rigid bronchoscopy was arranged for balloon dilatation of stenotic segment followed by TMC using the same method and dosage as described in previous cases. She reported initial improvements of symptoms which lasted for approximately two months. Repeated bronchoscopy revealed restenosis of the left main bronchus. She was then offered for evaluation for potential surgical treatment in which she declined. Fortunately, she remained stable on outpatient clinic follow‐ups despite physical limitations (Modified Borg Dyspnoea Scale 3 at rest: moderate; similar to initial presentation).

### Case 7

A 26‐year‐old lady had multilevel tracheobronchial stenosis (trachea and left and right main bronchus) secondary to EBTB. She had a background history of recurrent tuberculosis involving the gastrointestinal system in 2014 followed by PTB by 2019. While on treatment for PTB (after five months of medications), she presented with new‐onset worsening dyspnoea and reduced effort tolerance. With the diagnosis in mind, she underwent balloon dilatation of trachea and left main bronchus followed by application of TMC. The right main bronchus was totally obstructed. Her procedure was complicated by tracheal tear during balloon dilatation which fortunately was successfully managed conservatively without the need of emergency airway surgery. Post procedure, she reported improvements in symptoms which lasted for five months during her last clinical encounter with us.

## Discussion

Chung and Lee's classification described seven bronchoscopic subtypes of EBTB, namely: non‐specific bronchitic, oedematous‐hyperaemic, granular, ulcerative, caseating, tumorous, and fibrostenotic [9]. Fibrostenotic subtype represents a late fibrotic healing state characterized by airway luminal narrowing and destruction [9]. TSTB is debilitating and potentially life‐threatening as it may lead to asphyxia, respiratory failure, and even death [10]. The right main bronchus and right upper lobe segmental bronchi are the most common sites of EBTB while tracheal involvement is considered rare [8, 11, 12]. Common clinical presentations of TSTB include cough, wheezing, dyspnoea, and stridor [8]. TSTB should be suspected in patients with persistent or new‐onset respiratory symptoms despite currently on or have previously completed treatment for PTB. Two of our patients (case 1 and case 6) were initially treated as presumed adult‐onset bronchial asthma despite presenting with new‐onset shortness of breath while on treatment for PTB.

To date, the optimal treatment strategy of TSTB remains undefined. Open airway surgery for repair and/or resection of stenotic/malacic segment is often regarded as the gold standard treatment of choice for TSTB. In real‐life clinical practice, endoscopic interventions are meant to improve quality of life and to treat symptomatic TSTB that had formerly been considered either surgically untreatable (e.g. multilevel or long‐segment TSTB) or treatable only through extensive and often risky open airway surgeries. Many patients with TSTB have concurrent comorbidities including poor lung reserve due to extensive tuberculosis‐related pulmonary scarring and poor nutritional status, rendering open airway surgeries prohibitively dangerous.

Mitomycin‐C is an antineoplastic drug that acts as an alkylating agent by inhibiting DNA synthesis, replication, and transcription. At doses of lesser than 1 mg/mL, mitomycin‐C can inhibit cell division and fibroblast proliferation [13]. Originally licensed for the treatment of gastric and pancreatic carcinomas, TMC has been successfully used as an adjunct in the treatment of laryngotracheal stenosis secondary to granulomatosis with polyangiitis [14], idiopathic laryngotracheal stenosis [15], post‐intubation stenosis [15, 16], endobronchial sarcoidosis [17] as well as post lung transplant airway stenosis [18]. Recurrence of stenosis is one of the main drawbacks of endoscopic intervention and airway surgery [16]. Case reports have revealed that TMC can help to reduce the rate of recurrence among patients who underwent interventions for post‐intubation tracheobronchial stenosis [19, 20]. Besides, TMC appears to have a good safety profile. None of our patients developed complications directly related to TMC application. It is worth mentioning that pregnant women should not undergo TMC application due to potential harm to developing foetus. In addition, earlier studies by Kuo et al. and Kim et al. have demonstrated increased activities of matrix metalloproteinases‐1, interferon‐gamma, and transforming growth factor‐beta within EBTB lesions, thereby providing sound scientific background to support the use of TMC for TSTB [21, 22]. However, to date, little is known on whether patients with TSTB can ultimately benefit from TMC application in real‐life clinical practice. Our online PubMed search for “mitomycin‐C” and “tuberculosis” yielded only three relevant papers with a total of three TSTB patients successfully managed with endoscopic intervention involving TMC as an adjunct [6, 7, 8]. Currently, there are no studies to explore the optimal dosage and drug application time of TMC for patients with TSTB.

We attempted to explore whether the efficacy of TMC can be influenced by airway characteristics. All our patients with airway stenosis were successfully recanalized prior to application of TMC. A recent article by Lee et al. revealed that patients with bronchomalacia related to EBTB were 17 times more likely (odds ratio (OR): 17.179, *P* < 0.001) to suffer from recurrent symptoms post airway intervention compared to patients with pure TSTB without concurrent bronchomalacia [23]. Two of our patients with TSTB who developed rapid recurrence of symptoms post intervention had concurrent bronchomalacia (case 3 and case 6). Besides, airway intervention might be riskier in cases of bronchomalacia related to EBTB with reduced airway thickness and loss of airway cartilaginous support [24]. On the other hand, Shirit et al. demonstrated that TSTB patients needing more than one session of endoscopic airway intervention will subsequently require more definitive treatment in the form of either airway stent insertion or airway surgery [15]. Our second patient underwent more than five sessions of endoscopic balloon dilatation and laser cauterization prior to arrival to our centre. We postulate that multiple endoscopic interventions, together with the natural history and pathogenesis of TSTB, might have contributed to the formation of a thick fibrotic band surrounding the narrowed luminal opening in her case. The absorption of TMC into a thick fibrotic band is likely to be significantly lower compared to thin membranous concentric stenosis seen in other cases. In light of this, we hypothesize that TMC is more efficacious for TSTB patients with short concentric membranous stenotic segments without concurrent thick fibrotic band and/or bronchomalacia. We hope that further studies can help to establish the exact role and efficacy of TMC in the management of TSTB.

In conclusion, in the absence of large‐scale studies and guidelines, TSTB remains a challenging clinical entity to manage. With a good safety profile, TMC may be a useful adjunct in the management of short concentric membranous TSTB by reducing granulation tissue and preventing recurrence. Nevertheless, we hypothesize that TMC might be less efficacious among patients with long stenotic segments with concurrent bronchomalacia and/or thick fibrotic bands. We hope that more studies in the future can aid to bridge the current gaps in knowledge regarding the optimal management and role of TMC among this group of patients.

### Disclosure Statement

Appropriate written informed consent was obtained for publication of this case series and accompanying images.

### Author Contribution Statement

N.‐C. Huan and K.L. Ng contributed to the design and implementation of the research. N.‐C. Huan, K.L. Ng, M.Z. Nasaruddin, N.A. Muhammad, U.N. Daut, and J.A. Abdul Rahaman carried out the procedures mentioned. N.‐C. Huan and K.L. Ng wrote the manuscript. J.A. Abdul Rahaman supervised the project. All authors discussed the study and contributed to the final manuscript.
